# Impact of Environment and Social Gradient on *Leptospira* Infection in Urban Slums

**DOI:** 10.1371/journal.pntd.0000228

**Published:** 2008-04-23

**Authors:** Renato B. Reis, Guilherme S. Ribeiro, Ridalva D. M. Felzemburgh, Francisco S. Santana, Sharif Mohr, Astrid X. T. O. Melendez, Adriano Queiroz, Andréia C. Santos, Romy R. Ravines, Wagner S. Tassinari, Marília S. Carvalho, Mitermayer G. Reis, Albert I. Ko

**Affiliations:** 1 Centro de Pesquisas Gonçalo Moniz, Fundação Oswaldo Cruz, Ministério da Saúde, Salvador, Brazil; 2 Secretária Estadual de Saúde da Bahia, Salvador, Brazil; 3 Escola Nacional da Saúde Pública, Fundação Oswaldo Cruz, Ministério da Saúde, Rio de Janeiro, Brazil; 4 Universidade Federal Rural do Rio de Janeiro, Rio de Janeiro, Brazil; 5 Division of International Medicine and Infectious Diseases, Weill Medical College of Cornell University, New York, New York, United States of America; Universidad de Buenos Aires, Argentina

## Abstract

**Background:**

Leptospirosis has become an urban health problem as slum settlements have expanded worldwide. Efforts to identify interventions for urban leptospirosis have been hampered by the lack of population-based information on *Leptospira* transmission determinants. The aim of the study was to estimate the prevalence of *Leptospira* infection and identify risk factors for infection in the urban slum setting.

**Methods and Findings:**

We performed a community-based survey of 3,171 slum residents from Salvador, Brazil. *Leptospira* agglutinating antibodies were measured as a marker for prior infection. Poisson regression models evaluated the association between the presence of *Leptospira* antibodies and environmental attributes obtained from Geographical Information System surveys and indicators of socioeconomic status and exposures for individuals. Overall prevalence of *Leptospira* antibodies was 15.4% (95% confidence interval [CI], 14.0–16.8). Households of subjects with *Leptospira* antibodies clustered in squatter areas at the bottom of valleys. The risk of acquiring *Leptospira* antibodies was associated with household environmental factors such as residence in flood-risk regions with open sewers (prevalence ratio [PR] 1.42, 95% CI 1.14–1.75) and proximity to accumulated refuse (1.43, 1.04–1.88), sighting rats (1.32, 1.10–1.58), and the presence of chickens (1.26, 1.05–1.51). Furthermore, low income and black race (1.25, 1.03–1.50) were independent risk factors. An increase of US$1 per day in per capita household income was associated with an 11% (95% CI 5%–18%) decrease in infection risk.

**Conclusions:**

Deficiencies in the sanitation infrastructure where slum inhabitants reside were found to be environmental sources of *Leptospira* transmission. Even after controlling for environmental factors, differences in socioeconomic status contributed to the risk of *Leptospira* infection, indicating that effective prevention of leptospirosis may need to address the social factors that produce unequal health outcomes among slum residents, in addition to improving sanitation.

## Author Summary

Leptospirosis, a life-threatening zoonotic disease, has become an important urban slum health problem. Epidemics of leptospirosis now occur in cities throughout the developing world, as the growth of slum settlements has produced conditions for rat-borne transmission of this disease. In this prevalence survey of more than 3,000 residents from a *favela* slum community in Brazil, Geographical Information System (GIS) and modeling approaches identified specific deficiencies in the sanitation infrastructure of slum environments—open sewers, refuse, and inadequate floodwater drainage—that serve as sources for *Leptospira* transmission. In addition to the environmental attributes of the slum environment, low socioeconomic status was found to independently contribute to the risk of infection. These findings indicate that effective prevention of leptospirosis will need to address the social factors that produce unequal health outcomes among slum residents, in addition to improving sanitation.

## Introduction

At present, one billion of the world's population resides in slum settlements [1]. This number is expected to double in the next 25 years [1]. The growth of large urban populations which are marginalized from basic services has created a new set of global health challenges [2],[3]. As part of the Millennium Development Goals [4], a major priority has been to address the underlying poor sanitation and environmental degradation in slum communities which in turn, are the cause of a spectrum of neglected diseases which affect these populations [2],[3],[5].

Leptospirosis is a paradigm for an urban health problem that has emerged due to recent growth of slums [6],[7]. The disease, caused by the *Leptospira* spirochete, produces life-threatening manifestations, such as Weil's disease and severe pulmonary hemorrhage syndrome for which fatality is more than 10% and 50%, respectively [7]–[9]. Leptospirosis is transmitted during direct contact with animal reservoirs or water and soil contaminated with their urine [8],[9]. Changes in the urban environment due to expanding slum communities has produced conditions for rodent-borne transmission [6],[10]. Urban epidemics of leptospirosis now occur in cities throughout the developing world during seasonal heavy rainfall and flooding [6], [11]–[18]. There is scarce data on the burden of specific diseases that affect slum populations [2], however leptospirosis appears to have become a major infectious disease problem in this population. In Brazil alone, more than 10,000 cases of severe leptospirosis are reported each year due to outbreaks in urban centers [19], whereas roughly 3,000, 8,000 and 1,500 cases are reported annually for meningococcal disease, visceral leishmaniasis and dengue hemorrhagic fever, respectively, which are other infectious disease associated with urban poverty [20]–[22]. Case fatality (10%) from leptospirosis [19] is comparable to that observed for meningococcal disease, visceral leishmaniasis and dengue hemorrhagic fever (20%, 8% and 10%, respectively) in this setting [20],[23],[24]. Furthermore, leptospirosis is associated with extreme weather events, as exemplified by the El Niño-associated outbreak in Guayaquil in 1998 [25]. Leptospirosis is therefore expected to become an increasingly important slum health problem as predicted global climate change [26],[27] and growth of the world's slum population [1] evolves.

Urban leptospirosis is a disease of poor environments since it disproportionately affects communities that lack adequate sewage systems and refuse collection services [6],[10],[11]. In this setting, outbreaks are often due to transmission of a single serovar, *L. interrogans* serovar Copenhageni, which is associated with the *Rattus norvegicus* reservoir [6], [28]–[30]. Elucidation of the specific determinants of poverty which have led to the emergence of urban leptospirosis is essential in guiding community-based interventions which, to date, have been uniformly unsuccessful. Herein, we report the findings of a large seroprevalence survey performed in a Brazilian slum community (*favela*). Geographical Information System (GIS) methods were used to identify sources for *Leptospira* transmission in the slum environment. Furthermore, we evaluated whether relative differences in socioeconomic status among slum residents contributed to the risk of *Leptospira* infection, in addition to the attributes of the environment in which they reside.

## Methods

### Study site and population

**Figure 1:**
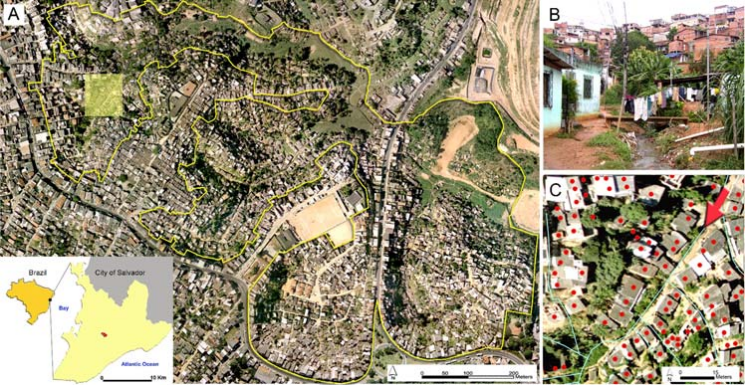
Slum community site in the city of Salvador, Brazil. (*A*) The yellow line in the aerial photograph is the boundary of the study site in the Pau da Lima community. The map in the bottom left corner shows the location of Salvador in Brazil and the study site (red) within the city. (*B*) Photograph of the typical environment at the community study site, which shows a valley in which households is situated and the proximity of households to open sewers and refuse. (*C*) Close-up view of the orthomap used to georeference households (red and black dots) and environmental attributes, such as open sewers (blue line) and refuse deposits, for the region marked as a yellow box in *Panel A*. The red arrow represents the direction from which the photograph in *Panel B* was taken.

The study was conducted in the Pau da Lima community (Figure 1A) which is situated in the periphery of Salvador, a city of 2,443,107 inhabitants [31] in Northeast Brazil. Pau da Lima is a region of hills and valleys, which was a sparsely inhabited area of Atlantic rain forest in the 1970s and subsequently transformed into a densely-populated slum settlement (Figure 1B) due to in-migration of squatters. In total, 67% of the population of Salvador and 37% of the urban population in Brazil reside in slum communities with equal or greater levels of poverty as that found in Pau da Lima [32],[33].

A study site was established which comprised of four valleys in an area of 0.46 km^2^ (Figure 1A). Active hospital-based surveillance found that the mean annual incidence of severe leptospirosis was 57.8 cases per 100,000 population at the study site between 1996 and 2001 (unpublished data). The study team conducted a census during visits to 3,689 households within the site in 2003 and identified 14,122 inhabitants. Households were assigned sequential numbers. A computer-based random number generator was used to select a list of 1,079 sample households from a database of all enumerated households. Eligible subjects who resided in sample households and had five or more years of age were invited to be a study participant. Subjects were enrolled into the study between April 2003 and May 2004 according to written informed consent approved by the Institutional Review Boards of the Oswaldo Cruz Foundation, Brazilian National Commission for Ethics in Research, and Weill Medical College of Cornell University.

### Household survey

The study team of community health workers, nurses and physicians conducted interviews during house visits and administered a standardized questionnaire to obtain information on demographic and socioeconomic indicators, employment and occupation, and exposures to sources of environmental contamination and potential reservoirs in the household and workplace. Responses reported by subjects were used to obtain information on race. The study team evaluated literacy according to the ability to read standardized sentences and interpret their meaning. Informal work was defined as work-related activities for which the subject did not have legal working documents. The head-of-household, defined as the member who earned the highest monthly income, was interviewed to determine sources and amounts of income for the household. Subjects were asked to report the highest number of rats sighted within the household property and the site of work-related activities. The study team surveyed the area within the household property to determine the presence of dogs, cats and chickens.

### Geographical Information System (GIS) survey

An ArcView version 8.3 software system (Environmental Systems Research Institute) database was constructed with georeferenced aerial photographs and topographic maps provided by the Company for Urban Development of the State of Bahia (CONDER). Photographs of the study site, which had a scale of 1∶2,000 and spatial resolution of 16cm, were taken in 2002. During the census, the study team identified households within the study site and marked their positions onto hard copy 1∶1,500 scale maps (Figure 1C), which were then entered into the ArcView database. A survey was conducted during the seasonal period of heavy rainfall between April and August 2003 to geocodify the location of open sewage and rainwater drainage systems. During three time points within this period, the study team mapped the sites of open accumulated refuse and measured the area of these deposits. Mean values for areas of refuse deposits were calculated and used for the analyses.

### Serological analysis

Sera were processed from blood samples collected from subjects during house visits. The microscopic agglutination test (MAT) was performed to evaluate for serologic evidence of a prior *Leptospira* infection [34]. A panel of five reference strains (WHO Collaborative Laboratory for Leptospirosis, Royal Tropical Institute, Holland) and two clinical isolates [6] were used which included *L. interrogans* serovars Autumnalis, Canicola and Copenhageni, *L. borgspetersenii* serovar Ballum, and *L. kirschneri* serovar Grippotyphosa. The use of this panel had the same performance in identifying MAT-confirmed cases of leptospirosis during surveillance in Salvador [6],[16] as did the WHO recommended battery of 19 reference serovars [34]. Screening was performed with serum dilutions of 1∶25, 1∶50 and 1∶100. When agglutination was observed at a dilution of 1∶100, the sample was titrated to determine the highest titer.

### Statistical methods

Information for subjects was double entered into an EpiInfo version 3.3.2 software system (Centers for Diseases Control and Prevention) database. Chi-square and Wilcoxon rank sum tests were used to compare categorical and continuous data, respectively, for eligible subjects who were and were not enrolled in the study. A P value ≤0.05 in two sided testing was used as the criterion for a significant difference. Preliminary analyses evaluated a range of MAT titers as criteria for prior *Leptospira* infection and found that the use of different cut-off values (1∶25–1∶100) identified similar associations with respect to the spatial distribution of seropositive subjects and risk factors for acquiring *Leptospira* antibodies. A titer greater or equal to 1∶25 was therefore used to define the presence of *Leptospira* antibodies in the final analyses. The presumptive infecting serovar was defined as the serovar against which the highest agglutination titre was directed [34]. Crude prevalence rates were reported since age and gender-adjusted values did not differ significantly from crude values. Ninety-five percent confidence intervals (CI) were adjusted for the cluster sampling of households.

Kernel density estimation analysis was performed with a range of bandwidths (10–120 meters) to evaluate smoothed spatial distributions of subjects with *Leptospira* antibodies and all subjects. The R version 2.4.1 statistical package (R Foundation for Statistical Computing) was used to obtain estimates which were adjusted for boundary effects. The ratio of the Kernel density estimators for subjects with *Leptospira* antibodies and all subjects was measured to determine the smoothed population-adjusted risk distribution. A digital terrain model of topographic data was used (ArcGIS 3D Analyst Extension software) to obtain continuous estimates of altitude for the study area. The distances, calculated in three-dimensional space, of households to nearest open drainage systems and refuse deposits were evaluated as proxies of exposure to these sources of environmental attributes. Elevation of households with respect to the lowest point in the valley in which they were situated was used as a surrogate for flood risk. Generalized additive models (GAM) [35] were used to evaluate the functional form of the association between continuous variables and the risk of acquiring *Leptospira* antibodies. When indicated, continuous variables were categorized in multivariate analyses according to the x-intercept value observed in the plots of fitted smoothed values.

We used Poisson regression [36] to estimate the effect of demographic, socioeconomic, household and workplace-related factors on the prevalence of *Leptospira* antibodies. A Bayesian inference approach was used which incorporated two random effects in order to account for overdispersion and cluster sampling within households. This approach has been used to estimate parameters in complex models [37] and is less sensitive to sparse data [38]. Standard non-informative prior distributions were used in models which were fitted with WinBUGS version 1.4.2 (MRC Biostatistics Unit). In multivariate analysis, all variables which had a P value below 0.10 in univariate analyses were included in the initial model. In order to address co-linearity among variables, we identified sets of covariates with the high Spearman correlation coefficients (>0.3 or <−0.3). Highly correlated variables were aggregated in a single variable when indicated, and evaluated in the model. The final model was obtained which used backward variable selection with an inclusion rule of P value <0.05.

## Results

Among 3,797 eligible residents from the slum community site, 3,171 (84%) were enrolled in the study. Study subjects had a higher proportion of females (56% of 3,171 subjects versus 37% of 626 subjects, respectively; P<0.05) and younger mean age (25.8±15.2 versus 28.1±14.6 years, respectively; P<0.05) than eligible residents who did not participate in the study. The kernel distribution of enrolled subjects according to place of residence was similar on visual inspection to that of residents who did not participate (data not shown). The majority (85%) of subjects were squatters who did not have legal title to their domiciles. Subjects belonged to mostly mixed (*pardo*, 66%) or black (28%) racial groups. Median household per capita income for study subjects was US$ 1.30 per day. Among the subjects, 76% had not completed elementary school education and 23% were illiterate. Among 2,077 subjects ≥18 years of age, 77% did not have formal employment and 35% engaged in informal work.

**Figure 2:**
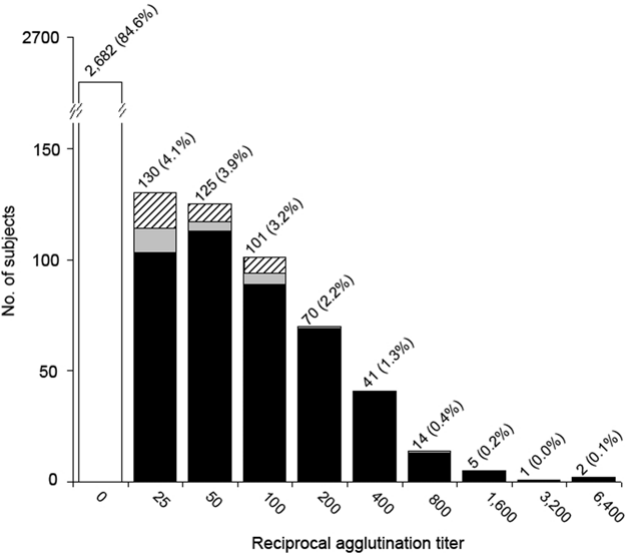
Distribution of reciprocal microscopic agglutination test titers for 3,171 subjects from the slum community site. Labels above the bars indicate the number of subjects (% of total), according to their highest reciprocal titer. The open bar represents seronegative subjects. Subjects with highest reciprocal titres against *L. interrogans* serovar Copenhageni, multiple serovars and serovars other than Copenhageni are shown as black bars, grey bars and crosshatched bars, respectively.

Among the 3,171 subjects, 489 had *Leptospira* agglutinating antibodies, as determined by the presence of MAT titer ≥1∶25 (Figure 2). Highest titers were directed against *L. interrogans* serovar Copenhageni in 436 (89.2%) of the 489 subjects with *Leptospira* antibodies. For the 22 subjects (4.5%) who had highest titers against two or more serovars, agglutination reactions recognized Copenhageni as one of the serovars. Copenhageni was the predominant serovar (88–100%) recognized for the range of highest reciprocal titers (Figure 2).

The overall prevalence of *Leptospira* antibodies was 15.4% (95% CI 14.0–16.8). The crude prevalence among enrolled subjects was not significantly different from the prevalence (15.9%, 95% CI 14.6–17.1) which was adjusted for the age and gender distribution of eligible subjects in the study population. Prevalence was highest among adolescents and adults (16.2% and 21.2% for age groups 15–24 and >44 years, respectively). However, 8.3% (95% CI 6.2–10.5) of children 5–14 years of age had evidence for a prior exposure to *Leptospira*. The prevalence was higher in males than females (17.8 versus 13.6%, respectively; PR 1.32, 95% CI 1.10–1.57) (Table 1). Similar associations with age and gender were observed when MAT titers of ≥1∶50 and ≥1∶100 were used to define subjects with *Leptospira* antibodies.

**Table 1:** Risk factors for *Leptospira* antibodies among subjects at the slum community site.

Variables	*Leptospira* antibodies		PR (95% CI)	
	Yes (n = 489)	No (n = 2,682)	Univariate^a^	Multivariate^b^
	**No. (%) or median (IQR)** c			
**Demographic**				
Age, years				
05–14	71 (15)	781 (29)	1.00	1.00
15–24	136 (28)	704 (26)	1.98 (1.47–2.61)	2.02 (1.50–2.69)
25–34	122 (25)	524 (20)	2.31 (1.71–3.07)	2.54 (1.86–3.41)
35–44	73 (15)	350 (13)	2.11 (1.50–2.88)	2.24 (1.59–3.08)
≥45	87 (18)	323 (12)	2.60 (1.88–3.51)	2.92 (2.10–4.00)
Male gender	247 (51)	1140 (43)	1.32 (1.10–1.57)	1.38 (1.14–1.64)
**Socioeconomic indicators**				
Black race^d^	169 (35)	724 (27)	1.35 (1.11–1.62)	1.25 (1.03–1.50)
Household per capita income, US$/day	1.14 (0.39–1.88)	1.30 (0.61–2.20)	0.91 (0.85–0.97)^e^	0.89 (0.82–0.95)^e^
Did not complete primary school	394 (81)	2018 (75)	1.32 (1.04–1.65)	-
**Household factors**				
Time of residence in household, years	8 (3–17)	7 (3–12)	1.02 (1.01–1.03)^e^	-
Level above lowest point in valley, meters	18.78 (8.59–31.05)	24.71 (13.00–36.04)	0.99 (0.98–0.99)^e^	-
Distance from an open sewer, meters	14.95 (7.34–31.00)	21.04 (8.99–38.11)	0.99 (0.99–1.00)^e^	-
Distance of household from an open sewer/lowest point in valley				
≥20 m/≥20 m	158 (32)	1198 (45)	1.00	1·00
≥20 m/<20 m	38 (8)	211 (8)	1.32 (0.89–1.83)	1.19 (0.81–1.67)
<20 m/≥20 m	73 (15)	360 (13)	1.46 (1.09–1.91)	1.30 (0.97–1.71)
<20 m/<20 m	220 (45)	913 (34)	1.68 (1.36–2.05)	1.42 (1.14–1.75)
Distance from an open refuse deposit, meters	60.59 (38.48–107.54)	64.90 (42.56–103.16)	1.00 (1.00–1.00)^e^	-
<20 meters from open refuse deposit	51 (10)	174 (6)	1.53 (1.12–2.02)	1.43 (1.04–1.88)
Vegetation^f^	373 (76)	1,822 (68)	1.45 (1.17–1.79)	-
Reservoirs present in household				
Sighting of >2 rats	256 (52)	1039 (39)	1.60 (1.33–1.91)	1.32 (1.10–1.58)
Dog	231 (47)	1028 (38)	1.36 (1.14–1.62)	-
Chicken	227 (46)	988 (37)	1.40 (1.17–1.66)	1.26 (1.05–1.51)
Cat	106 (22)	406 (15)	1.44 (1.15–1.77)	-
**Work-related exposures**				
Informal work	157 (32)	637 (24)	1.42 (1.17–1.71)	-
Contact with contaminated environment^g^	83 (17)	284 (11)	1.57 (1.22–1.96)	-
Risk occupation^h^	49 (10)	127 (5)	1.90 (1.37–2.51)	-

a Univaritate prevalence ratios (PR) and 95% confidence intervals (CI) are shown for variables which were significant (P<0.05) in the univariate analyses.

b Multivariate PR and 95% CI are shown for covariates which were included in the final best fit Poisson regression model.

c Numbers and percentages are shown for categorical variables. Median and interquartile range (IQR) are shown for continuous variables of per capita household income, time of residence in study household, level above lowest point in valley and distance from an open sewer and refuse deposit.

d Data is missing for two non-infected subjects.

e PR and 95% CI are shown for continuous data.

f Data is missing for one infected and two non-infected subjects.

g Reported exposure to mud, refuse, flooding water or sewage water in the workplace.

h Occupation as construction worker, refuse collector or mechanic, which is associated with a workplace environment characterized by high rat infestation.

**Figure 3:**
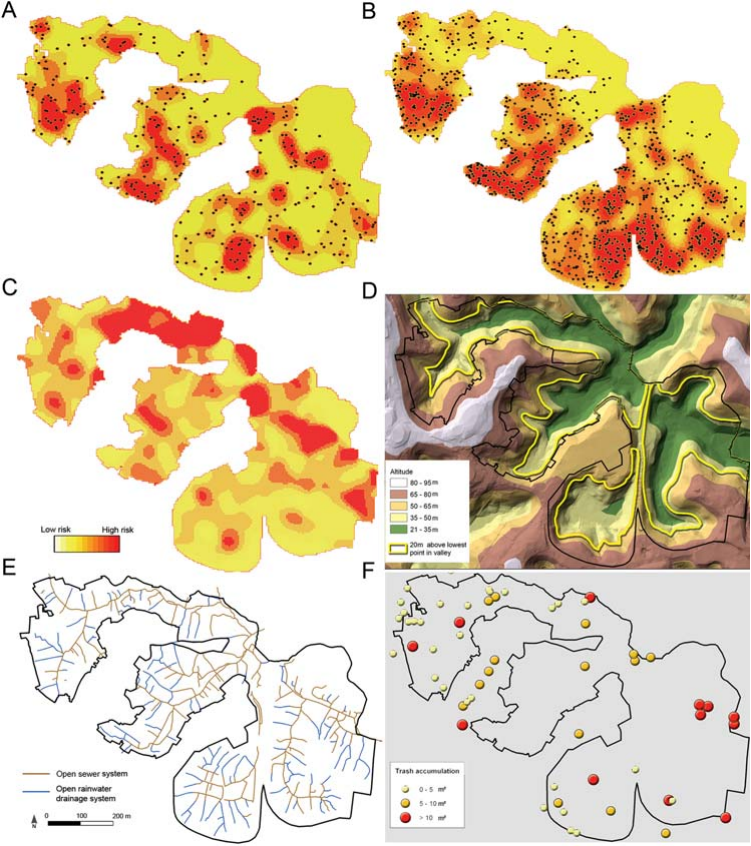
Spatial distribution of subjects with *Leptospira* antibodies and all enrolled subjects, according to place of residence, and environmental attributes of the community site. *Panels A* and *B* show the smoothed Kernel density distribution of subjects with *Leptospira* antibodies (N = 489) and all (N = 3,171) subjects, respectively, according to place of residence. The yellow-to-red gradient represents increasing density in smoothing analyses which used 40 meters as the bandwidth. Black circles show the location of subject households. *Panel C* shows the distribution of the population-adjusted Kernel density estimator for subjects with *Leptospira* antibodies which was calculated as the ratio of the estimators for subjects with *Leptospira* antibodies and all subjects. *Panel D* shows a topographic map generated by the digital terrain model. The yellow line is the level that is 20 meters above the lowest point in the four valleys within the community site. *Panels E* and *F* show the distribution of, respectively, open rainwater and sewage drainage systems and accumulated refuse according to size (m^2^).

Panels A and B in Figure 3 show smoothed spatial distributions of subjects with *Leptospira* antibodies and all subjects, respectively, according to place of residence. The population-adjusted distribution (Figure 3C) showed that risk of acquiring *Leptospira* antibodies clustered in areas occupied by squatters at the bottom of valleys (Figure 3D). Similar spatial distributions were observed in analyses that used higher titer values to define subjects with *Leptospira* antibodies (Figure S1).

Univariate analysis found the risk of acquiring *Leptospira* antibodies to be associated with increasing age, male gender, indicators of low socioeconomic level, occupations that entail contact with contaminated environments, informal work, time of residence in the study household, and environmental attributes and the presence of reservoirs in the household (Table 1). Significant risk associations were not found for formal employment and reported sighting of rats in the workplace environment. Open rainwater drainage structures and refuse deposits were distributed throughout the site; yet open sewers were more frequently encountered at the bottom of valleys (Figure 3). The distance of household to the nearest open sewer was a risk factor, whereas a significant association was not observed for distance to an open rainwater drainage system.

**Figure 4:**
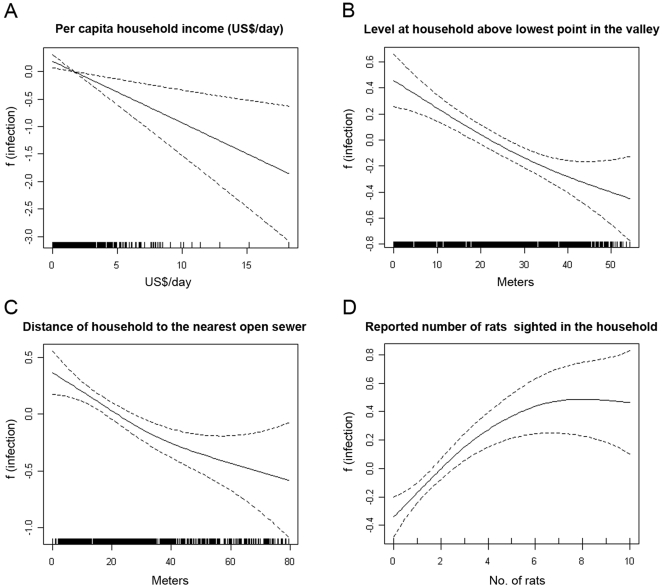
Generalized additive models (GAM) of the association between the risk of acquiring *Leptospira* antibodies and continuous variables of (*A*) per capita daily household income, (*B*) level of household in meters above the lowest point in valley, and (*C*) distance in meters to the nearest open sewer, and (*D*) reported number of rats sighted in the household environment. The coefficient, f(infection), in the GAM model is a measure for the risk of acquiring *Leptospira* antibodies. In *Panels A, B, C* and *D*, the x axis intercept values, where f(infection) equals zero, were US$1.70/day, 22 meters, 22 meters and 2 rats, respectively.

GAM analysis showed that the risk of acquiring *Leptospira* antibodies had an inverse linear association with the distance of the subject's household to an open sewer and elevation of the household from the lowest point in the valley, a proxy for flood risk (Figure 4). Increased risk was observed among subjects who resided less than a threshold distance of 20 meters to these attributes (Figure 4B and C). The risk of acquiring *Leptospira* antibodies had an inverse non-linear association with distance of the subject's household to an open refuse deposit (results not shown). We explored a range of dichotomization criteria and found significant risk associations when subjects resided less than 20 meters from an open refuse deposit (Table 1). This association was not influenced by the size of the refuse deposit. Subjects who reported sighting two or more rats in the household environment had increased risk of acquiring *Leptospira* antibodies (Figure 4D). Household per capita income had an inverse linear association with the presence of *Leptospira* antibodies (Figure 4A). Of note, the distance of the household to an open sewer was highly correlated (Spearmen correlation coefficient = 0.71) with household elevation (Figure S2A) since open sewers drain into the bottom of valleys. An aggregate variable, distance of household located less than 20 meters from an open sewer and lowest point in a valley, was therefore used to examine the association between open sewer and flood-related exposure and infection risk (Table 1). In contrast household per capita income was not highly correlated (Spearmen correlation coefficient = 0.16) with the elevation of the household (Figure S2B).

Multivariate analyses found that the risk for acquiring *Leptospira* antibodies was associated with exposures in the household environment and not in the workplace setting (Table 1). Subjects who resided less than 20 meters from an open sewer and the lowest point in the valley had a 1.42 times (95% CI 1.14–1.75) increased risk for acquiring *Leptospira* antibodies than those who lived 20 meters or more from these attributes. Residence less than 20 meters from accumulated refuse was associated with a 1.43 times (95% CI 1.04–1.88) increased risk. Sighting of two or more rats and presence of chickens, a marker for rat infestation, in the household were significant reservoir-associated risk factors. After controlling for age, gender and significant environmental exposures, indicators of low socioeconomic level, household per capita income (PR 0.89 for an increase of US$1.00 per day, 95% CI 0.82–0.95) and black race (PR 1.25, 95% CI 1.03–1.50) were risk factors for acquiring *Leptospira* antibodies (Table 1).

## Discussion

Efforts to identify interventions for urban leptospirosis have been hampered by the lack of population-based information on transmission determinants. In this large community-based survey of a slum settlement in Brazil, we found that 15% of the residents had serologic evidence for a prior *Leptospira* infection. The prevalence rate of *Leptospira* antibodies in the study slum community was similar to that (12%) found in a city-wide survey performed in Salvador [39]. Risk factors for acquiring *Leptospira* antibodies were associated with exposures in the household environment. Interventions therefore need to target the environmental sources of transmission - open sewers, flooding, open refuse deposits and animal reservoirs - in the places where slum inhabitants reside. After controlling for the influence of poor environment, indicators of low socioeconomic status were found to be independently associated with the risk of acquiring *Leptospira* antibodies. This finding suggests that in slum communities with overall high levels of absolute poverty, relative differences in socioeconomic level contribute to unequal outcomes for leptospirosis.

Leptospirosis has been traditionally considered an occupational disease, since work-related activities are frequently identified as risk exposures [9]. However slum inhabitants reside in close proximity to animal reservoirs and environmental surface waters which contain *Leptospira* [10]. We previously found that *Leptospira* infection clusters within households in slum communities in Salvador [40]. In this study, we found that after controlling for confounding, significant risk exposures were those associated with the household environment rather than workplace. As a caveat, interview-elicited responses were used to evaluate work-related exposures since GIS surveys were not performed at the sites where subjects worked. It is possible that slum residents may have had work-related risk exposures which were not detected by our survey. Nevertheless, our findings support the conclusion that the slum household is an important site for *Leptospira* transmission and provides the rationale for interventions that target risk exposures in this environment.

The study's findings indicate that the domestic rat was the principal reservoir for *Leptospira* transmission in the study community. Highest agglutination titers among 89% of the subjects were directed against *L. interrogans* serovar Copenhageni, the serovar associated with the *R. norvegicus* reservoir. Reported sighting of rats is considered to be an unreliable marker of rat infestation. However we found that the number of rats sighted by residents was correlated with their risk of acquiring *Leptospira* antibodies (Figure 4D), indicating that rat sightings may be a useful marker of infection risk in slum communities where inhabitants are accustomed to the presence of rats. Although dogs were not found to be a risk factor, detailed investigations of *Leptospira* carriage in urban reservoirs need to be performed. Of note, the presence of chickens in households was a risk factor, although they in of themselves are not reservoirs. This association may reflect a rat-related exposure not accounted for by reported sightings, since rats are attracted to chicken feed and waste. Raising chickens is a widespread practice in slum communities-48% (519) of the 1079 study households raised chickens. Control of rodent reservoir populations may therefore need to incorporate measures that directly address this practice.

Our findings confirm hypotheses raised by previous ecologic studies [6],[10],[11] that infrastructure deficiencies related to open sewers, flooding and open refuse deposits are transmission sources for leptospirosis in the slum environment. Furthermore, there appears to be defined areas of risk associated with open sewers and refuse deposits, which serve as habitats and sources of food for rats. Home range radius of the domestic rat varies from 30–150 meters [41],[42], but home range use decreases from the centre to the edge. GAM analysis demonstrated that slum residents had a positive risk for acquiring *Leptospira* antibodies when households were situated within 20 meters from open sewers and refuse deposits. In addition, infection risk increased as distances from an open sewer or refuse deposit decreased, suggesting that households which are situated closer to these foci have a higher degree of environmental contamination with *Leptospira* and inhabitants of these households are exposed to higher inoculum doses during infection. Molecular approaches to quantify *Leptospira* in environmental samples [10] will be useful in answering this question and guiding recommendations for environmental decontamination and barrier control measures which can be implemented in slum communities.

In addition, GAM analysis found that residents had positive risk for *Leptospira* infection when their households were situated within 20 meters from the lowest point in the valley (Figure 4B). In Salvador [6],[12],[16],[40] and other urban centers [11],[13],[15],[17],[18], outbreaks of leptospirosis occur during heavy rainfall and flooding events. Slum communities are built on the poor land quality and often in areas susceptible to frequent flooding. At the study site and other slum settlements in Salvador, the water table rises up to one meter during flooding events because of inadequate rainwater drainage and blockage of drainage systems with silt and refuse. The finding that subjects had increased infection risk when their households were located within 20 meters from the lowest point in the valley suggests that this distance was a proxy for the degree of contact which residents encounter flood-related exposures in the peri-domiciliary environment.

We found that in addition to attributes of the environment where slum inhabitants reside, low per capita household income and black race, an indicator of health inequality in Brazil [43],[44], were independent risk factors for *Leptospira* infection. The social gradient in health is a widespread phenomenon [45],[46]. Our findings, although not unexpected, are noteworthy since they suggest that differences in status contribute to unequal health outcomes in a slum community where the household per capita income was less than US$1 per day for 44% of the inhabitants. Although errors in the measurement of risk exposures and residual confounding were a possibility, the strength of the association indicates a role for social determinants in *Leptospira* transmission. These factors may relate to risky behaviors, such as cleaning open sewers after flooding events, or limited use of protective clothing which reduce the risk of abrasions that facilitate entry of the *Leptospira* spirochete [47]. Low status and lack of access to amenities and social support are features of disadvantaged communities [45] which conceivably influence risk behaviors for leptospirosis. Further research is needed to evaluate the role of social factors such that effective interventions, including health education, can be implemented at the community level.

A limitation of our study was the cross-sectional design which used serologic evidence for a prior *Leptospira* infection as the outcome. The MAT is the standard assay used in prevalence surveys [9], yet there is not an established titer criterion for defining seropositive reactions. We previously found that a MAT titer of ≥1∶25 was a specific marker for prior *Leptospira* infection among slum residents from Salvador and when applied, identified household clustering of infection risk [40]. In this study, cutoff titers from 1∶25 and above identified similar risk associations. In Salvador, leptospirosis is due to transmission of a single agent, *L. interrogans* serovar Copenhageni [6],[28]. Titers of 1∶25, as well as higher titers, were directed against this serovar (Figure 1), indicating that this cutoff was a specific and more sensitive criteria for identifying prior infections in a region where a single serovar agent is circulating. In the study, there were more men and younger subjects among non-participating subjects than participating subjects. Crude prevalence was not different from the prevalence of *Leptospira* antibodies which was adjusted by the age and gender distribution of the overall study population, indicating that differences between participating and non-participating subjects may not have introduced a significant bias in the estimates. Infections may have occurred up to five years prior to the survey since agglutinating antibodies may persist for this period [48],[49]. Major interventions to improve basic sanitation were not implemented in the study community, yet the possibility that environmental exposures were modified over time can not be excluded. Migration may have affected our ability to estimate prevalence and risk associations. An on-going cohort investigation of subjects enrolled in this study found that the annual out-migration rate is approximately 12% (unpublished data). The study's findings therefore need to be confirmed in prospective studies.

We found that *Leptospira* transmission was due to the interaction of factors associated with climate, geography and urban poverty. Since the study was performed in a single community in Salvador, Brazil, our findings may not be generalizable to other slum settings. However, a large proportion of the world's slum population resides in tropical climates similar to that in Salvador. Moreover, similar conditions of poverty and environmental degradation encountered at the study site (Figure 1B) are found in many slum settlements. In Brazil, 37% of the urban population resides in slums with equal or greater levels of poverty as found in the study community [33]. Our findings may therefore be relevant to other slum communities where leptospirosis is endemic and have increasing significance as global climate change [26],[27] and growth of the world's slum population occur in the future [1],[33].

The infrastructure deficiencies which were found to be transmission factors for *Leptospira* in this study can be readily addressed by improving sanitation in slum communities. Investment in sanitation is a cost-effective health intervention [50],[51]. In Salvador, a city-wide sanitation program (*Bahia Azul*) was recently shown to have a major beneficial impact for diarrheal disease [52]. However, as frequently encountered with large-scale sanitation projects, the *Bahia Azul* program did not provide coverage to the study community and many of the slum settlements in the city's periphery. Equitable access to improved sanitation is therefore essential in reducing the burden of the large number of environmentally-transmitted infectious diseases, including leptospirosis, which affects slum populations. Furthermore, the finding that the social gradient within slum communities, in addition to the unhealthy environment, contributes to the risk of *Leptospira* infection suggests that prevention of urban leptospirosis will need to combine approaches for improving sanitation with approaches that identify and address the social determinants which produce unequal health outcomes.

## Supporting Information

Figure S1 Smoothed Kernel density distribution of subjects with microscopic agglutination test titres of ≥1∶25 (*A*), ≥1∶50 (*B*) and ≥1∶100 (*C*), according to place of residence at the study site. The yellow-to-red gradient represents increasing density in smoothing analyses which used 40 meters as the bandwidth.
(2.61 MB TIF)

Figure S2 Spot plots of the relationship between elevation of household level from the lowest point in valley and distance of the household to the nearest open sewer (*A*) and household per capita daily income (*B*). Closed and open dots represent houses with at least one seropositive subject and without a seropositive subject, respectively.
(1.02 MB TIF)

Alternative Language Abstract S1 Abstract translated into Portuguese by Dr. Guilherme Ribeiro.
(0.03 MB DOC)
