# Supplementary figures and images for: Impact of Environment and Social Gradient on Leptospira Infection in Urban Slums
Source: PLoS Negl Trop Dis. 2008 Apr 23;2(4):e228. doi: 10.1371/journal.pntd.0000228 (PMC2292260; doi:10.1371/journal.pntd.0000228)

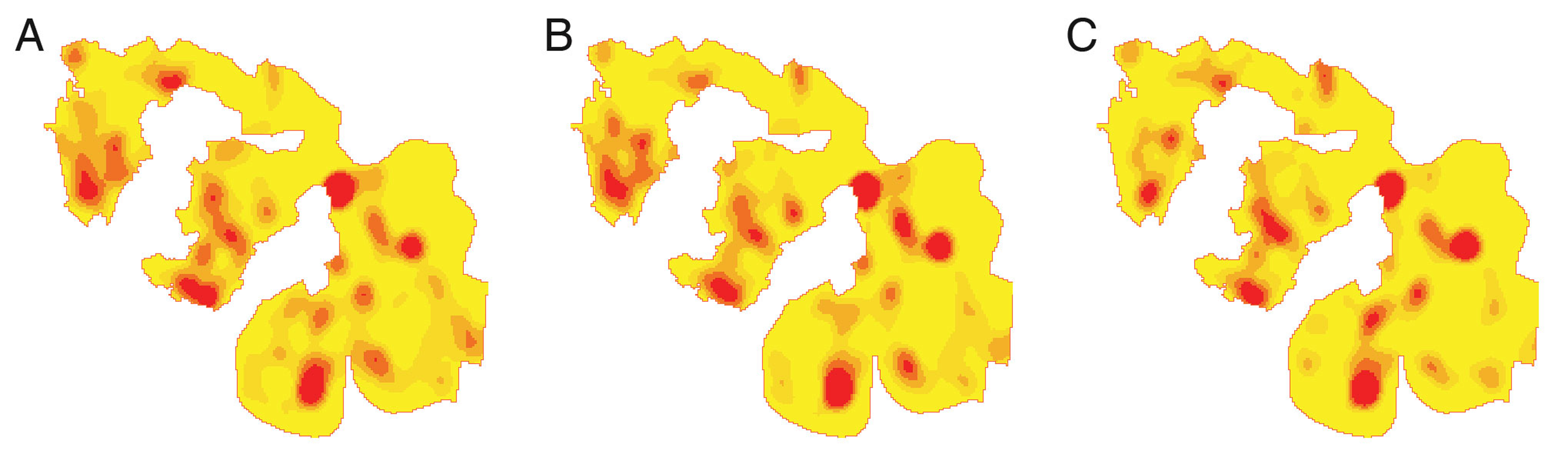

Supplement: Figure S1 — Smoothed Kernel density distribution of subjects with microscopic agglutination test titres of ≥1∶25 (A), ≥1∶50 (B) and ≥1∶100 (C), according to place of residence at the study site. The yellow-to-red gradient represents increasing density in smoothing analyses which used 40 meters as the bandwidth. (2.61 MB TIF) [file pntd.0000228.s001.tif]

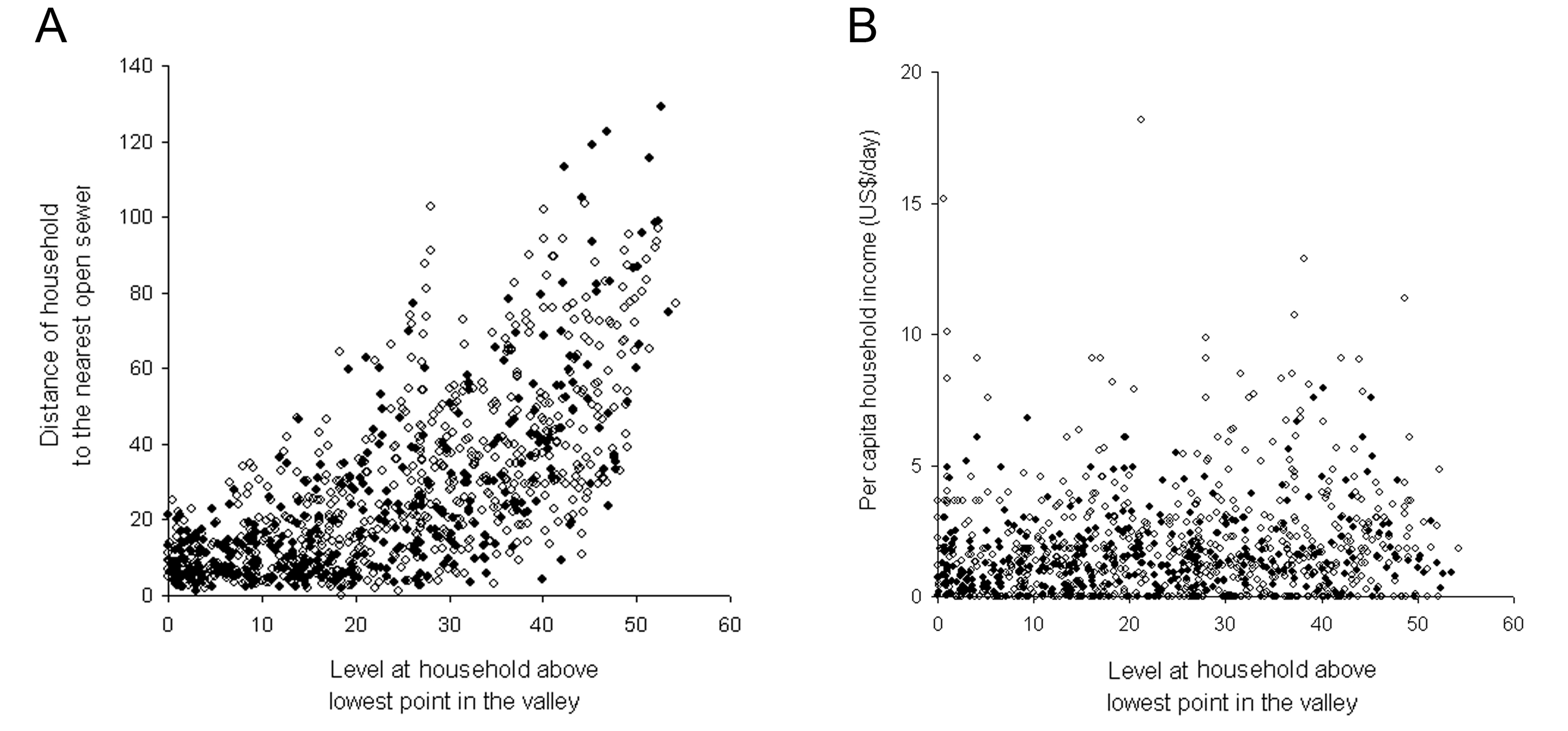

Supplement: Figure S2 — Spot plots of the relationship between elevation of household level from the lowest point in valley and distance of the household to the nearest open sewer (A) and household per capita daily income (B). Closed and open dots represent houses with at least one seropositive subject and without a seropositive subject, respectively. (1.02 MB TIF) [file pntd.0000228.s002.tif]
