# Supplementary material for: Impact of Environment and Social Gradient on Leptospira Infection in Urban Slums
Source: PLoS Negl Trop Dis. 2008 Apr 23;2(4):e228. doi: 10.1371/journal.pntd.0000228 (PMC2292260; doi:10.1371/journal.pntd.0000228)
Supplement: Alternative Language Abstract S1 — Abstract translated into Portuguese by Dr. Guilherme Ribeiro. (0.03 MB DOC) [file pntd.0000228.s003.doc]

**Abstract for 08-PNTD-RA-0029 translated in Portuguese by Dr. Guilherme Ribeiro**

***Introdução:*** Leptospirose tornou-se um problema de saúde urbano devido à expansão das favelas em todo mundo. Entretanto, a falta de informações populacionais sobre os determinantes de transmissão tem dificultado a identificação de intervenções para o controle da leptospirose urbana. Este estudo tem como objetivos estimar a prevalência da infecção pela *Leptospira* e identificar fatores de risco para infecção em uma favela.

***Métodos e Resultados:*** Nós conduzimos um inquérito de base comunitária em 3.171 residentes de uma favela em Salvador, Brasil. Anticorpos aglutinantes contra *Leptospira* foram usados como um marcador de infecção prévia. Modelos de regressão de Poisson avaliaram a associação entre a presença de anticorpos contra *Leptospira* e atributos ambientais obtidos através de Sistema de Informação Geográfica, indicadores sócio-econômicos e exposições de risco individuais. A prevalência de anticorpos contra *Leptospira* foi de 15,4% (intervalo de confiança [IC] de 95%, 14,0-16,8). Os domicílios dos indivíduos com anticorpos contra *Leptospira* agrupavam-se áreas de invasão no fundo dos vales da favela. A aquisição de anticorpos contra *Leptospira* foi associada a fatores ambientais do peri-domicílio como residência em regiões com esgotos aberto e risco para alagamento (razão de prevalência [RP] 1,42, IC 95% 1,14-1,75) e proximidade a acúmulo de lixo (1,43, 1,04-1,88), observação de ratos (1,32, 1,10-1,58), e presença de galinhas (1,26, 1,05-1,51). Além disso, baixo nível sócio-econômico e raça negra (1,25, 1,03-1,50) foram fatores de risco independentes. Para cada acréscimo de US$1 na renda domiciliar per capita diária observou-se uma redução de 11% (IC 95% 5-18%) no risco de infecção.

***Conclusões:*** Deficiências na estrutura de saneamento onde habitantes de favelas residem foram identificados como fontes ambientais de transmissão da *Leptospira*. Mesmo após controlar para estes fatores ambientais, diferenças no nível sócio-econômico contribuíram para o risco de infecção pela *Leptospira*. Estes achados indicam que além de melhorias no saneamento, medidas de prevenção efetivas para leptospirose deveriam ser dirigidas para a redução das diferenças sociais que produzem desfechos de saúde desiguais entre residentes de favelas.
